# Fine mapping with epigenetic information and 3D structure

**DOI:** 10.1007/s00281-021-00906-4

**Published:** 2022-01-12

**Authors:** Gisela Orozco

**Affiliations:** 1 Centre for Genetics and Genomics Versus Arthritis, Division of Musculoskeletal and Dermatological Sciences, School of Biological Sciences, Faculty of Biology, Medicine and Health, The University of Manchester, AV Hill Building, Oxford Road, Manchester, M13 9LJ UK; 2 NIHR Manchester Biomedical Research Centre, Manchester University NHS Foundation Trust, Manchester Academic Health Science Centre, Manchester, UK

**Keywords:** Autoimmune disease, Genome-wide association studies, Fine mapping, Functional genomics, Epigenetics, Chromatin conformation

## Abstract

Since 2005, thousands of genome-wide association studies (GWAS) have been published, identifying hundreds of thousands of genetic variants that increase risk of complex traits such as autoimmune diseases. This wealth of data has the potential to improve patient care, through personalized medicine and the identification of novel drug targets. However, the potential of GWAS for clinical translation has not been fully achieved yet, due to the fact that the functional interpretation of risk variants and the identification of causal variants and genes are challenging. The past decade has seen the development of great advances that are facilitating the overcoming of these limitations, by utilizing a plethora of genomics and epigenomics tools to map and characterize regulatory elements and chromatin interactions, which can be used to fine map GWAS loci, and advance our understanding of the biological mechanisms that cause disease.

## Introduction

Complex diseases arise from a combination of genetic and environmental factors. Genome-wide association studies (GWAS) have identified thousands of genetic regions, or loci, that contain single nucleotide polymorphisms (SNPs) associated with complex diseases [1]. For autoimmune diseases, multiple, large-scale genetic association studies have been carried out, including GWAS, fine mapping studies, follow-up studies and meta-analyses, contributing to the identification of a large proportion of the heritability of these diseases.

The ultimate goal of genetic studies is to improve human health, by enabling a better understanding of the biological mechanisms that lead to disease. This, in turn, can help identify novel therapeutic targets for autoimmune diseases for which there is a lack of specific treatments, or where a large proportion of patients do not respond appropriately to available treatments, such as psoriatic arthritis (PsA), Crohn’s disease, ankylosing spondylitis (AS) or rheumatoid arthritis (RA), among many others. Indeed, several studies have shown that drug development programs with incidental genomic support had a higher rate of developmental success [2–5]. In this regard, a coding variant in ﻿*TYK2*, which has a protective effect in multiple sclerosis (MS), AS, ulcerative colitis (UC) and Crohn’s disease, leads to a weaker response of CD4 + T helper 1 and 17 type cells to proinflammatory signals [6], which suggested that reducing the activity of TYK2 could be a potential therapeutic option for these diseases. There are now multiple clinical trials testing TYK2 inhibitors in several autoimmune diseases [7].

Furthermore, biological mechanisms revealed by GWAS can suggest compounds suitable for repurposing, which involves the use in a new disease of a drug approved for treatment of a different condition and allows cheaper and quicker translation implementation of treatments into the clinic compared to de novo drug discovery [8]. For example, the monoclonal antibodies ustekinumab and risankizumab, first used to treat psoriasis, target components of the interleukin-23 (IL-23) signalling pathway. One of the most robust GWAS signals in Crohn’s disease is a missense mutation on the IL-23 receptor (*IL23R*) gene, which inspired the exploration of the above-mentioned treatments in Crohn’s disease [9]. These biologics have now been proven to be successful in the treatment of Crohn’s and in other diseases that also present genetic associations with genes in the IL-23 pathway such as PsA and AS [10–14].

Genetics also have the potential to aid precision medicine approaches to target the best treatments available to specific groups of patients who are more likely to respond appropriately or avoid particular treatments in patients who are predicted to present adverse reactions [15]. For example, certain HLA alleles have been shown to be correlated with treatment response in psoriasis [16] and rheumatoid arthritis (RA) [17]. In addition, genetic risk scores (GRS) can help identifying those at highest risk of developing disease. Although they are less powerful in predicting traits with low prevalence, disclosure of high genetic risk to individuals in the clinic can lead to behavioural changes to minimize exposure to environmental risk factors [15]. The ongoing investment in long-term prospective cohort studies that include genetic data combined with detailed electronic health records, such as the UK Biobank [18], will help advance the field of precision medicine [19].

However, despite these exciting and promising advances, the full potential of GWAS for clinical translation has not been achieved yet, due to several caveats that make the interpretation of GWAS variants difficult [20]. First, each individual GWAS-associated variant acts as a signpost or “tag” for a haplotype containing multiple neighbouring SNPs that are inherited together in a block, i.e. they are in high linkage disequilibrium (LD). Therefore, GWAS alone cannot distinguish the causal variants underpinning the association from that of the other variants in LD with them. Second, over 90% of GWAS variants map to non-coding regions of the genome, and therefore, they do not directly affect the coding sequence of a gene, making it challenging to identify the biological mechanism by which they cause disease [21, 22]. Third, in many cases, it is unclear which are the causal genes, since GWAS loci often contain multiple genes in the vicinity or map at very large distances from coding genes. And finally, we lack a complete understanding of the context, i.e. the cell types and stimuli, under which disease associated variants have an effect.

Fine mapping using statistical methods, for example, Bayesian approaches, credible SNP sets and trans-ethnic meta-analyses, have been successful in ﻿prioritizing genetic variants for further study [23, 24], but to overcome the limitations listed above and fully realize the potential of GWAS to understand disease biology, follow-up functional studies are needed. In this review, different functional genomics approaches to fine map GWAS loci and prioritize causal genes in autoimmune diseases will be discussed, with examples illustrating how these methods have been used to translate genetic findings into functional understanding of etiopathological mechanisms of autoimmune disease.

## Functional genomics

The non-coding portion of the human genome is thought to play a pivotal role in the highly complex process that is the regulation of gene expression, which is mediated by regulatory elements. Regulatory elements are short, non-coding functional DNA sequences that can regulate transcription of their target genes and contain binding sites for regulatory proteins. There are several classes of regulatory elements, such as enhancers, promoters, insulators or silencers, that are characterized by slightly different features, such as specific histone modifications or binding or particular transcription factors [25, 26]. As above mentioned, the vast majority of GWAS-associated variants maps to non-coding regions of the genome, and they seem to be predominantly enriched in cell type-specific enhancers [27].

**Fig. 1:**
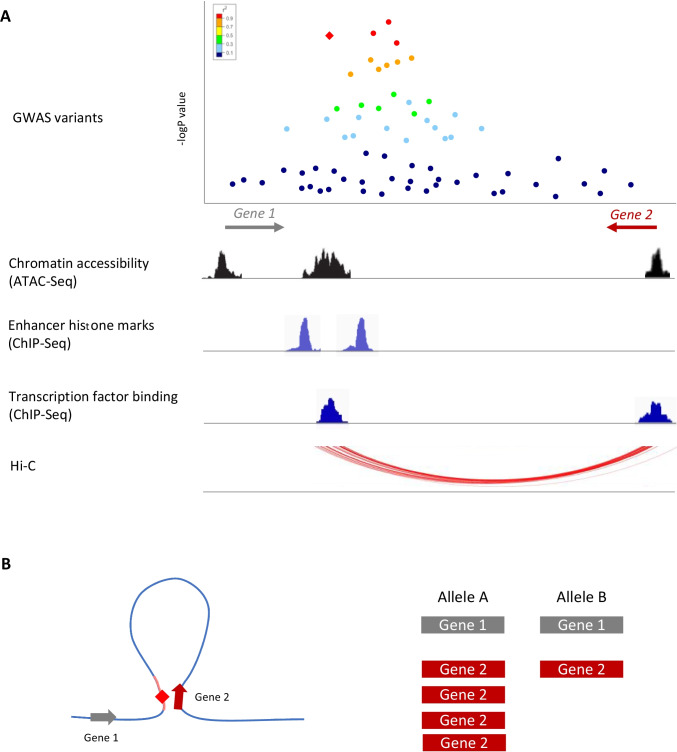
Schematic representation of how functional genomics data can be used to functionally interpret GWAS loci. **A** Fine mapping using epigenetic information and colocalization: amongst the most strongly associated SNP within a disease GWAS locus (red dots), only one (red diamond) overlaps an active enhancer, suggesting that this variant may be the causal SNP. **B** The disease enhancer containing the risk variant, although closer in the linear conformation to *Gene 1*, interacts with *Gene 2* through chromatin looping. In addition, the disease SNP is an eQTL for *Gene 2* but has no effect on the expression of *Gene 1*, suggesting that *Gene 2* may be the causal gene at this locus

The mapping and characterization of regulatory elements such as enhancers using functional genomics has therefore been used extensively to functionally interpret GWAS signals across all complex traits [28, 29] (Fig. 1). Functional genomics involves the use of genomic data to investigate gene expression and function at genome-wide scale, usually by applying high-throughput methods (Table 1). Regulatory elements generally map to regions of open chromatin, are flanked by nucleosomes with histone modifications that are associated with active transcription, are bound by specific transcription factors and are likely to physically come into contact with their target genes through chromatin looping [28]. Major large-scale international projects, like Encode [30], Roadmap [31] or the ﻿IHEC consortium [32], have used techniques to detect such features at the genome-wide scale (like ATAC-Seq, ChIP-Seq and Hi-C, Table 1) in a wide range of cell and tissue types, which has become an invaluable resource in the post-GWAS era.

**Table 1:** Summary of the genomics, epigenomics and transcriptomics techniques most commonly used to interpret GWAS signals

Type	Technique	Description
Transcription	RNA-seq, GRO-cap, CAGE [33, 34]	Whole-transcriptome RNA sequencing can identify transcription of active enhancers (enhancer RNA or eRNAs)
Transcription	eQTLs [35]	Correlation between genetic variation and levels of gene expression
Chromatin accessibility	MNase-seq, DNase-seq, ATAC-seq [36–38]	High-throughput detection of open chromatin by micrococcal nuclease digestion, cleavage by DNase I or library construction using the hyperactive transposase Tn5, respectively, followed by next-generation sequencing (NGS)
Histone marks	ChIP-seq, Cut&Run [39, 40]	Detection of post-translational histone modifications by immunoprecipitation with specific antibodies, e.g. enhancer or promoter-associated histone modifications such as H3K4me1 or H3K27ac, followed by NGS
Protein binding	ChIP-seq, Cut&Run [39, 40]	Detection of DNA bound regulatory proteins and transcription factors by immunoprecipitation with specific antibodies, e.g. RNA polymerase II or NFκB, followed by NGS
3D proximity	Chromatin conformation capture (3C) methods, i.e. Hi-C, Capture Hi-C (CHi-C), HiChIP [41–43]	Family of methods to detect looping and spatial organization of DNA. The chromatin is digested with enzymes, and then interacting regions are re-ligated together. The resulting products are sequenced and analysed to quantify the frequency of interactions

## Fine mapping using epigenetic information and colocalization

Understanding the mechanism by which SNPs lead to changes in molecular phenotypes in disease relevant tissues is required for the identification of causal variants in risk loci identified by GWAS. The majority of GWAS SNPs are thought to disrupt regulatory elements, and therefore, these mechanisms most commonly involve alteration of gene expression levels, disruption of transcription factor binding and perturbations of enhancer activity, in the form of alterations in chromatin accessibility and histone modifications [44]. Therefore, numerous functional fine-mapping methods are based on the colocalization of GWAS variants in high LD with functional annotations such as promoters and enhancers using data from large-scale projects like Encode or Roadmap, described above. If a variant, or variants, within a locus overlaps active functional elements in tissues of interest, they will be more likely to be causal than variants in LD that do not [45] (Fig. 1). It is important to note that although many fine-mapping methods assume that only one SNP per locus contributes to disease, it has been shown that multiple variants within a single locus can play a role through additive or epistatic effects, which can context cell type specific [46].

Applying this colocalization approach, Trynka et al. examined 15 epigenetic chromatin marks using ChIP-seq data from 14 immune cell types from the ENCODE project [47]. After overlapping with all associated SNPs for 31 complex traits, they found that H3K4me3 was the most cell type-specific chromatin mark. Indeed, there was an enrichment of risk SNPs in peaks for this histone mark in cell types that are keys for disease biology. For example, in RA, GWAS SNPs were enriched in H3K4me3 peaks in CD4 + Treg cells. Interestingly, when the analysis was repeated using newly defined index SNPs from the dense genotyping fine-mapping study Immunochip [48], instead of index SNPs from the largest RA GWAS at that time [49], the significance of the enrichment for CD4 + Treg cells increased. In addition, an RA-associated locus tagged by rs13119723, an SNP mapping to an intron of the gene of unknown function *KIAA1109* and containing multiple variants in LD spanning over 500 Kb, was fine mapped to a single SNP mapping to a H3K4me3 peak specific to CD4 + Treg cells thought to be involved in the regulation of *IL2* expression. These results demonstrate how colocalization approaches of GWAS and functional annotations can inform both the most relevant cell types implicated in disease and the fine mapping of associated SNPs to identify causal variation.

In another seminal study, Farh et al. aimed at fine-mapping GWAS loci from 21 autoimmune diseases by developing an algorithm, Probabilistic Identification of Causal SNPs (PICS), that estimates the probability that an individual SNP is a causal variant given the haplotype structure and observed pattern of association at the locus, followed by integration of transcription and cis-regulatory element annotations from the NIH Roadmap and similar data [22]. They estimated that 60% of causal variants map to immune enhancers, but only 10–20% directly alter recognizable transcription factor binding motifs. Multiple methods have been developed recently to enhance statistical fine mapping with functional annotations, significantly improving the identification of causal variants. For example, Weissbrod et al.developed a functionally informed method, PolyFun, that specifies prior causal probabilities for subsequent fine-mapping methods, providing > 20% power increase over nonfunctionally informed fine-mapping methods [50].

A number of other studies have also showed that overlap of disease variants with functional annotations can identify tissue-specific enrichments [51, 52]. In addition to cell type specificity, disease variants can have an effect on phenotype in specific cell states or under specific stimulatory conditions. Soskic et al. stimulated T cells, and macrophages with an array of 13 different cytokines found that immune disease-associated variants are enriched in chromatin regions that are open and active in early rather than late activation of memory CD4^+^ T cells [53].

The most complete human epigenome reference to date, EpiMap (epigenome integration across multiple annotation projects), was recently assembled, by compiling 10,000 epigenomic maps from the main large-scale consortia studies such as ENCODE, Roadmap and IHEC, including ChIP-Seq data for numerous histone marks and transcription factors, open chromatin data from DNAse-seq and ATAC-seq experiments, across 800 samples from multiple cell types and tissues [54]. These datasets were used to define chromatin states, enhancers and target genes, which were then used to annotate 30,000 genetic loci associated with 540 traits from the GWAS catalogue, predicting key disease tissues, causal SNPs enriched in enhancers and candidate target genes for each locus.

Transcription factor binding profiling has also been used to functionally interpret non-coding variation. IMPACT is a recently developed genome annotation method that identifies regulatory elements defined by cell-state-specific TF binding profiles, learned from 515 chromatin and sequence annotations [55]. When integrated with RA GWAS data, it was found that the top 5% of CD4 + Treg regulatory elements identified by IMPACT capture 85.7% of RA genetic heritability, outperforming methods that ignore ﻿differential functionality of effector cell states. This further strengthens the importance of selecting the right tissues and cell types in functional studies. This annotation method has also been proven to be useful in the identification of causal variants that are common for different populations, despite the presence of different LD patterns at associated loci, which improves the trans-ancestry portability of polygenic risk scores [56].

On the other hand, high-throughput protein-DNA binding assays, rather than predictions, have been used to elucidate the biological function of non-coding variation. ﻿Resources such as the systematic assessment of the binding of 270 human transcription factors to 95,886 non-coding variants in the human genome using one of such methods, SNP-SELEX, further facilitates the understanding of the pathways involved in disease [57]. When applied to T2D, this study found that SNPs that demonstrate differential binding of TFs were highly enriched in the set of SNPs that had been previously reported as likely causal. The T2D-associated SNP rs7578326, which overlaps a candidate enhancer and had been linked to the *IRS1* gene by long-range chromatin interactions in HepG2 cells, was found to affect binding of the liver-specific transcription factor CEBPB. Using CRISPR interference, the enhancer was silenced in HepG2 cells, which resulted in significantly reduced expression of *IRS1*.

In addition to enabling the identification of regulatory elements across the genome, the outputs of high-throughput functional genomics methods described above can be utilised to assess whether genetic variants present allele-specific effects. The allele-specific abundance of sequencing reads can help us generate hypothesis about the biological function of disease variants﻿ (quantitative trait loci mapping, QTL). This type of study can profile traits such as chromatin accessibility (caQTL), histone marks (hQTL), exon splicing (sQTL) gene expression (eQTL) or protein expression (pQTL).

Several studies have shown that many caQTLs overlap TF binding sites and motifs, and a subset of them colocalize with eQTLs and GWAS variants, which suggests that SNPs that map to these loci influence GWAS traits by altering chromatin accessibility [58–62]. Likewise, the presence of hQTLs that affect enhancer-associated histone ChIP-Seq peaks like H3K27ac or H3K4me1 further suggests that these variants have an effect on cell type-specific enhancer activity [52]. Pelikan et al. identified numerous hQTLs in lymphoblastoid cell lines derived from systemic lupus erythematosus (SLE) patients, which were enriched in autoimmune disease risk haplotypes and influenced gene expression variability compared with non-hQTL variants in strong LD, suggesting that this type of data can be used in fine-mapping efforts [63].

## Gene prioritization using expression quantitative trait loci (eQTLs)

The examples described in the previous section illustrate how functional genomics annotations have identified thousands of enhancers that are disrupted by GWAS variants. However, only a handful of these have been confidently linked to their target genes, limiting the biological knowledge of the pathways that are altered in disease that we can extrapolate from these studies. Due to the robustness of RNA-sequencing technologies, eQTL studies represent a common approach to prioritize potential causal genes at risk loci.

Several methods have been used to integrate eQTL maps with GWAS data, from just ﻿assessing whether GWAS variants were also significant eQTLs, to more sophisticated colocalization statistical analyses [29]. Early studies that overlapped the most strongly associated GWAS variants at a given locus with the top eQTL within the same variants described how risk SNPs are more likely to be eQTLs than random non-associated SNPs [64, 65]. Examples of how this type of study has been used to pinpoint disease genes include the analysis conducted by Westra et al. where an SLE-associated SNP was also found to be an eQTL for the TF *IKF1*. This eQTL also affected expression of a number of other genes that are in turn regulated by IKF1 [66].

More accurate statistical colocalization methods were later developed, to account for the fact that the large amount of eQTLs present in the genome can result in a high degree of false positives due to chance, multiple genes can be affected by the same variant (pleiotropy), and that the top eQTL SNP in a locus may not always be the same or in tight LD with the top GWAS SNP [67–70]. It is also important to know that eQTLs can be context specific and can change with stimulation with pathogens or IFN-γ, for example [71–73].

Large-scale projects that characterize the genetic factors underlying gene expression across tissues and cell types provide a highly valuable resource. The Genotype-Tissue Expression (GTEx) consortium represents one of the most comprehensive eQTL resources [74]. GTEx’s approach is to perform both DNA sequencing and multi-tissue RNA-seq across many post-mortem donor samples, to identify genetic variants that are correlated with the expression levels of genes across the whole genome. The third and final phase of the project was recently completed, with a dataset of 838 donors and 15,201 samples across 49 tissues, identifying more than 4.2 million eQTLs which were enriched in disease-associated variants from the GWAS catalogue [75].

Another noteworthy large-scale eQTL mapping effort relevant for autoimmune diseases is eQTLGen, which includes whole blood-derived expression from 31,684 individuals [76]. One strength of this study is the capability to identify trans-effects, which are harder to detect than cis-eQTLs. Forty-seven GWAS traits for which at least four independent variants affected the same gene in trans were identified; in SLE, 13 genes in the SLE interferon signature were affected by at least three SLE-associated genetic variants each.

GTEx and eQTLGen data are generated from bulk tissue samples, and given the cell type specificity of the regulation of gene expression, their sample heterogeneity may confound mechanistic follow-up of GWAS loci. Kim-Hellmuth et al. have developed a computational deconvolution method to estimate the proportion of different cell types in GTEx tissues, so then cell type information can be accounted for in eQTL analyses [77]. But, ideally, disease relevant cell types, such as individual immune cell populations isolated from blood, can be more informative for the colocalization of eQTLs with GWAS variants in autoimmunity. The DICE study studied 13 different immune cell types from 91 donors and discovered that a large fraction (41%) of the 12,254 genes for which eQTLs were identified showed a strong *cis*-association with genotype only in a single cell type [78]. Of note, the ImmuNexU study identified immune cell type- and disease-specific eQTLs, by studying 28 distinct immune cell subsets from patients with 10 different immune-mediated diseases and healthy individuals in Japanese population. Most large-scale eQTL studies such as GTEx, eQTLGen or DICE have been generated in European donors; the ImmuNexU study revealed eQTL variants in East Asian populations that had not been detected in European populations, highlighting the need for ancestry-matched eQTL databases for GWAS fine mapping and improvement of functional annotation [79].

There are many other eQTL studies that have made their datasets publicly available, which represents an incredibly valuable resource. Recently, the eQTL catalogue provided uniformly processed gene expression and splicing QTLs from all available public studies, facilitating the widespread use of these resources [80].

## Gene prioritization using chromatin 3D structure

Enhancers and their target genes, which might be distant from one another in the linear DNA primary structure, are brought close together by chromatin loops. Therefore, the genes affected by GWAS variants that disrupt regulatory elements can be identified by characterizing 3D chromatin structure in relevant cell types using methods like Hi-C [81, 82]. Hi-C is based on chromosome conformation capture (3C), a method to detect looping and spatial organization of DNA. The chromatin is digested with enzymes, and interacting regions are re-ligated together. The resulting products are sequenced and analysed to quantify the frequency of interactions (Table 1). In addition to chromatin loops, Hi-C data can be used to map higher order chromatin structures, such as active/inactive A/B compartments and topologically associating domains (TADs) representing large domains that display a marked regulatory potential, that can help us understand the chromatin context of disease associated loci.

It has been shown that abnormal 3D chromatin organization can lead to disease by rewiring interactions between genes and regulatory elements [83] and that there is a direct relationship between the strength of chromatin interaction, DNA activity, and gene expression, where small changes in interaction intensity mediated by disease-associated regulatory genetic variation lead to large functional effects in gene expression [84, 85]. In addition, recent evidence has demonstrated that SNPs can influence chromatin interaction strength, along with accessibility and gene expression [86] and, interestingly, that T1D risk haplotypes demonstrate a greater level of interactions and gene activity in mice [87].

In a seminar paper, Javierre et al. applied a technique based on Hi-C that incorporates a ﻿sequence capture step to enrich for interactions that involve specific regions of interest, capture Hi-C (CHi-C), to identify interacting regions of all known promoters in the human genome, in 17 human primary hematopoietic cell types [88]. They found that chromatin interactions between promoters and promoter-interacting regions (PIRs) are highly cell type specific. Interestingly, PIRs were found to be significantly enriched in active chromatin regions and eQTLs. Similarly, PIRs were also enriched in GWAS variants associated with autoimmune diseases in lymphoid cells, and the data was subsequently used to identify putative causal genes in these traits. Of note, multiple genes such as *RP4-753F5.1*, *CD101*, *TTF2* and *TRIM45* were prioritized as RA candidate genes at the 1p13.1 GWAS locus, which had been initially assigned to *CD2* due to proximity.

Alternatively, Hi-C libraries can be enriched by immunoprecipitation using an antibody targeting a protein or histone modification of interest, in a method called HiChIP. Recently, Chandra et al.used H3K27ac HiChIP to map interactions between eQTLs overlapping active *cis*-regulatory elements and their target genes in five immune cell types, increasing our understanding the mechanism by which disease risk variants exert their effects on gene expression, in a cell-specific manner [89].

Other studies have successfully used a similar approach to link associated variants to their target genes in autoimmune diseases. A study targeting all GWAS loci for RA, T1D, PsA and JIA (as opposed to capturing gene promoters) in T and B cell lines also showed that chromatin interactions can be cell type specific [90]. Multiple putative causal genes were identified, and it was found that some disease-associated SNPs do not interact with the nearest gene but with other candidate genes that can be situated several megabases away and that regions associated with different autoimmune diseases can interact with each other and the same promoter. In addition, this data proved to be useful in the identification of drugs that could be repositioned to treat rheumatic diseases [91].

Other studies have highlighted the importance of non-immune cell types in autoimmune diseases. For example, stromal cells, such as synovial fibroblasts (FLS), play an important role in RA, but functional genomics datasets in this cell type were lacking. A recent study aimed at characterizing DNA architecture, 3D chromatin interactions, DNA accessibility and gene expression in FLS samples from RA patients; it was found that FLS account for up to 24% of RA heritability and 10% of the RA-associated regions contain SNPs located within enhancers that are exclusive to FLS, which suggests an additional, independent role of FLS in driving the genetic risk of developing RA [92]. The same study found that TNF stimulation of FLS alters the organization of topologically associating domains, chromatin state and the expression of putative RA causal genes such as *TNFAIP3* and *IFNAR1*. Therefore, future functional studies in non-immune cell types that contribute to the pathogenesis of autoimmune diseases will likely advance fine mapping of GWAS loci.

These studies and many others [93, 94] exemplify how 3D chromatin structure can aid the functional interrogation of GWAS loci. However, Hi-C methods often lack resolution to characterize individual enhancer-promoter interactions at sufficient depth since interaction maps are constrained by the restriction enzyme used, typically *HindIII*. This can be only partially solved with the use of multiple restriction enzymes that digest the genome into smaller fragments, like the strategy used in commercially available kits (Arima Genomics). A new analysis method, based on a Bayesian sparse variable selection approach, was developed to fine map chromatin contacts [95]. On the other hand, exciting novel technological advances, such as the development of a new 3C technique called ﻿Micro-Capture-C, are starting to pave the way to increase resolution of chromatin interactions at the base-pair level [96]. Although this method cannot be applied at the genome wide level yet, it provides an unprecedented level of detail about how gene expression is regulated by enhancers containing disease-associated variants and will facilitate fine mapping of GWAS loci.

## Conclusions

In the past decade, advances in large-scale methods for the functional characterization of autoimmunity risk loci have enabled a deeper understanding of the genetics underlying disease. However, the complexity of the regulatory networks that are affected by disease variants and the ever increasing number of genetic variants to dissect [97] means that further work is needed to accelerate clinical translation of genetics findings.

In this regard, the high degree of context specificity of some disease-associated regulatory elements means that bulk chromatin profiling methods may be limited for the detection of regulatory elements that are active in very specific cell types or developmental/stimulatory conditions. This challenge will likely be overcome by recently developed single cell (sc) methods like scATAC-seq [98], scChIP-seq [99] and scHi-C [100], among others. These methods are likely to reveal even more complex layers to the dysregulation of gene expression that characterize complex autoimmune diseases and will probably replace bulk assays in the future. For example, the single-cell eQTLGen Consortium has recently been established to investigate the consequences of disease-associated genetic variants in specific immune cell types at the gene expression level [101]. Furthermore, multi-sector initiatives such as The Accelerating Medicines Partnership (AMP) program will advance our knowledge of the key cell types that need to be targeted to understand the genetic basis of autoimmune disease. Indeed, using single cell transcriptomics, distinct cell states that are expanded in RA have been identified as part of phase I of the AMP program [102].

Functional investigation of GWAS variants using epigenomics and 3D chromatin structure is extremely valuable in the generation of hypotheses as to which SNPs within a disease locus might be causal, the regulatory mechanism they might be disrupting and in which cell or tissue type, and their likely causal or target gene/s. These hypotheses then need to be experimentally validated. Although still somewhat limited by effect size, efficiency and implementation in disease relevant primary cells, clustered regularly interspaced short palindromic repeats (CRISPR)-based methods have become the gold standard for the functional dissection of GWAS loci in recent years [103]. The use of CRISPR-based technologies and massively parallel reporter assays (MPRAs) to functionally interpret GWAS signals is reviewed extensively elsewhere in this issue of *Seminars in Immunopathology* [104].

Finally, meaningful integration of the vast wealth of functional data and knowledge that has been generated in the past few years is essential for elucidating biological networks that are altered in disease and for fulfilling the potential of GWAS for drug development and repositioning. Initiatives such as Open Targets [105, 106] and bioinformatics pipelines like EpiMap [54], IMPACT [55] and others [45, 107, 108] provide frameworks to prioritize potential targets by integrating GWAS data with genomic features, disease ontologies and network connectivity. However, data integration still currently remains a challenge, and further research is needed in this area.

These exciting developments, together with future advances and the use of patient-derived biological material and electronic health records, will further our understanding of complex autoimmune diseases, assist drug development and enable precision medicine, delivering on the promise of GWAS for patient benefit.

## Data Availability

Not applicable.
